# Facilitating spice recognition and classification: An image dataset of Indian spices

**DOI:** 10.1016/j.dib.2024.110936

**Published:** 2024-09-21

**Authors:** Sandip Thite, Deepali Godse, Kailas Patil, Prawit Chumchu, Alfa Nyandoro

**Affiliations:** a Vishwakarma University, Pune, India; b Bharati Vidyapeeth's College of Engineering for Women, Pune, India; c Kasetsart University, Sriracha, Thailand; d Regent University, Virginia Beach, VA, USA

**Keywords:** Agriculture, Culinary art, Food science, Indian spices, Spice classification

## Abstract

This data paper presents a comprehensive visual dataset of 19 distinct types of Indian spices, consisting of high-quality images meticulously curated to facilitate various research and educational applications. The dataset includes extensive imagery of the following spices: Asafoetida, Bay Leaf, Black Cardamom, Black Pepper, Caraway Seeds, Cinnamon Stick, Cloves, Coriander Seeds, Cubeb Pepper, Cumin Seeds, Dry Ginger, Dry Red Chilly, Fennel Seeds, Green Cardamom, Mace, Nutmeg, Poppy Seeds, Star Anise, and Stone Flowers. Each image in the dataset has been captured under controlled conditions to ensure consistency and clarity, making it an invaluable resource for studies in food science, agriculture, and culinary arts. The dataset can also support machine learning and computer vision applications, such as spice recognition and classification. By providing detailed visual documentation, this dataset aims to promote a deeper understanding and appreciation of the rich diversity of Indian spices.

Specifications Table

**Table:** 

Subject	Agronomy, Food Science, Crop Science
Specific subject area	Indian Spices
Data format	Raw
Type of data	Image
Data collection	This dataset offers a comprehensive collection of 10,991 high-quality images of Indian spices captured at Market Yard, Gultekadi, Pune, Maharashtra, India. It focuses on 19 of the most significant spice categories in India: Asafoetida, Bay Leaf, Black Cardamom, Black Pepper, Caraway Seeds, Cinnamon Stick, Cloves, Coriander Seeds, Cubeb Pepper, Cumin Seeds, Dry Ginger, Dry Red Chilly, Fennel Seeds, Green Cardamom, Mace, Nutmeg, Poppy Seeds, Star Anise, and Stone Flowers. Images are meticulously organized into separate folders for each category. For each spice category, images were taken from various angles and under different lighting conditions, ensuring a rich and diverse collection. Each individual spice image was captured under controlled lighting conditions to ensure consistent image quality. Images are saved in JPG format and resized to a uniform resolution of 810×1080 pixels for efficient storage and processing. The images were renamed sequentially for clear organization within the dataset. This meticulous approach in capturing and organizing the images ensures that the dataset is a valuable resource for both academic research and practical applications within the food industry.
Data source location	Market Yard, Gultekadi, Pune, Maharashtra 411,037. State : Maharashtra, Country- India. Latitude-18.491236855969703, Longitude-73.87033507123967
Data accessibility	Repository name: Indian Spices Image Dataset Data identification number: 10.17632/vg77y9rtjb.3 Direct URL to data : https://data.mendeley.com/datasets/vg77y9rtjb/3

## 1 Value of the Data

• *Comprehensive and Diverse*: The dataset includes 19 distinct categories of spices, each represented through a broad range of images taken from multiple angles and under different lighting conditions. This comprehensive approach ensures a rich diversity that can cater to various analytical and research needs.
• *First Open-Access Dataset*: This dataset is the first openly accessible collection of Indian spices.
• *Support for Machine Learning and Image Recognition*: The extensive and meticulously curated collection of 10,991 images provides an excellent dataset for training machine learning models and image recognition algorithms. The variety of angles and lighting conditions enhances the robustness and accuracy of these models.
• *Diverse Applications in Research and Development*: This dataset can be used in various research fields, including computer vision, agricultural studies, and culinary sciences. It offers a valuable resource for developing applications that require precise identification and classification of spices.
• *Culinary and Food Industry Insights*: The dataset serves as a practical tool for the food industry, aiding in quality control, inventory management, and the development of digital catalogs for spices. It can also assist culinary professionals and enthusiasts in identifying and understanding different spices.
• *Academic and Educational Resource*: The dataset is an invaluable resource for academic purposes, providing rich material for coursework, research projects, and thesis work related to food science, agriculture, and computer science.
• *Standardized Image Quality and Organization*: The images are captured under controlled lighting conditions and resized to a uniform resolution, ensuring consistent quality. The clear and sequential naming convention enhances the ease of use and accessibility for various users, facilitating efficient data processing and analysis.
• *Documentation of Indigenous Knowledge***:** The dataset captures the traditional uses of Indian spices for culinary and medicinal purposes across various communities, aiding in the preservation of knowledge that might otherwise be lost to younger generations.
• *Identification of Medicinal Properties***:** The dataset's ethnobotanical studies are vital for identifying spices with potential pharmacological properties, potentially leading to the development of new drugs and therapeutic agents.
• *Preservation of Cultural Heritage***:** The dataset records the use of spices in traditional practices, helping preserve cultural heritage and documenting diverse culinary and medicinal practices across Indian communities.

Overall, this dataset's high-quality images and detailed organization make it a significant asset for advancing research, innovation, and practical applications in multiple fields.

## 2 Background

While extensive research has been conducted on plants and vegetables [[1], [2], [3], [4], [5], [6], [7], [8], [9], [10], [11], [12], [13]], there is a noticeable lack of comprehensive datasets focused on Indian spices, which are crucial to both culinary practices and agricultural studies. Indian cuisine is renowned for its rich and diverse use of spices, commonly referred to as “Masala” in regional languages. These spices are integral to the flavor, aroma, and color of Indian dishes, playing a crucial role in culinary traditions. Beyond their culinary applications, Indian spices hold significant cultural, medicinal, and economic value, making them an essential part of daily life and heritage.

The 19 spices featured in this dataset—Asafoetida, Bay Leaf, Black Cardamom, Black Pepper, Caraway Seeds, Cinnamon Stick, Cloves, Coriander Seeds, Cubeb Pepper, Cumin Seeds, Dry Ginger, Dry Red Chilly, Fennel Seeds, Green Cardamom, Mace, Nutmeg, Poppy Seeds, Star Anise, and Stone Flowers—are among the most essential and widely used in Indian cooking. Each spice has its own unique properties and applications, contributing to the complexity and depth of Indian cuisine.

Masalas have a storied history in India, with their use dating back thousands of years. They are mentioned in ancient texts and have been a part of traditional Ayurvedic medicine [10]. Spices such as turmeric, ginger, and black pepper were highly valued in ancient trade, leading to the establishment of trade routes and influencing global cuisine [11]. Indian households have traditionally used a variety of spices not only for their flavors but also for their purported health benefits, such as aiding digestion, boosting immunity, and possessing anti-inflammatory properties.

India is one of the largest producers and exporters of spices in the world. The spice trade contributes significantly to the Indian economy, providing livelihoods to millions of farmers and workers. Spices like black pepper, cardamom, and cumin are among the most exported, reaching markets worldwide. The global demand for Indian spices continues to grow, driven by the increasing popularity of Indian cuisine and the recognition of spices' health benefits.

The use of masalas varies across different regions of India, each with its own distinctive spice blends and culinary techniques. For example, the garam masala blend commonly used in North India differs significantly from the sambar powder used in South Indian cuisine. This regional diversity adds to the richness of Indian culinary traditions and provides a wide array of flavors and aromas.

With the growing interest in food technology, machine learning, and image recognition, there is an increasing demand for high-quality, well-organized datasets that can support research and development in these areas. This Indian spices dataset was created to meet this demand, providing a valuable resource for scientists, researchers, and professionals in various fields. The dataset's meticulous organization and diverse range of images ensure that it can be used for a wide array of applications, from developing machine learning models for spice identification to conducting studies on the visual and physical characteristics of spices.

Captured at Market Yard, Gultekadi, Pune, Maharashtra, this dataset reflects the authentic and diverse nature of Indian spices. Pune, being one of the major market hubs in India, offers an ideal location for capturing a representative collection of spices commonly used across the country. The controlled lighting conditions and uniform resolution of the images ensure consistent quality, making the dataset a reliable and valuable tool for both academic and practical applications.

By providing a comprehensive collection of spice images, this dataset aims to support and enhance the study and utilization of Indian spices in various technological and culinary contexts. It stands as a significant contribution to the fields of food science, machine learning, and digital imaging, promoting further innovation and research in these areas.

### 2.1 Real-World usage examples of spices

The Indian Spices Dataset has potential applications across various fields, providing valuable insights and practical solutions. Below are some examples of how similar datasets have been successfully utilized in real-world scenarios:

### 2.2 Culinary research and recipe development

Researchers and chefs have used datasets like this to analyze the flavor profiles, nutritional content, and health benefits of spices. For instance, studies on the antioxidant properties of spices have led to the development of new recipes that prioritize both flavor and health. Such research is essential in promoting the use of spices in modern culinary practices and enhancing the nutritional value [14].

### 2.3 AI-powered spice identification and sorting

In the spice processing industry, machine learning models trained on spice datasets have been developed to automatically identify and sort spices. This technology increases efficiency and accuracy, reducing the need for manual labor. Companies have adopted these AI solutions to enhance their production processes [15].

## 3 Data Description

This dataset comprises a comprehensive collection of 10,991 high-quality images showcasing 19 distinct types of Indian spices, captured at Market Yard, Gultekadi, Pune, Maharashtra, India. Each spice category—Asafoetida, Bay Leaf, Black Cardamom, Black Pepper, Caraway Seeds, Cinnamon Stick, Cloves, Coriander Seeds, Cubeb Pepper, Cumin Seeds, Dry Ginger, Dry Red Chilly, Fennel Seeds, Green Cardamom, Mace, Nutmeg, Poppy Seeds, Star Anise, and Stone Flowers—is meticulously organized into separate folders. For each category, multiple images were taken from various angles and under different lighting conditions, ensuring a rich and diverse collection. The images were captured under controlled lighting conditions to maintain consistent quality. All images are saved in JPG format and have been resized to a uniform resolution of 810 × 1080 pixels for efficient storage and processing. Additionally, images within each category folder were renamed sequentially for clear and organized identification. This meticulously curated dataset serves as a valuable resource for machine learning, image recognition, culinary studies, and various research and development endeavors within the food industry. All the images were taken in JPG format because it's a widely used image type, perfect for datasets. Its efficient lossy compression helps reduce file size while still maintaining good quality. JPG is also compatible across various platforms, supports millions of colors, and is optimized for fast processing. Its versatility and relevance in real-world applications make it an ideal choice for large datasets, especially in fields like computer vision.

The datasetʼs images are of high quality, with a resolution set at 72 dots per inch (dpi), ensuring clear and detailed visual representation of the spices. In version 2 of the dataset on Mendeley Data, we have added a file titled “Metadata-Indian_Spices Dataset Files Description,” which contains detailed metadata information [1].

**Fig. 1:**
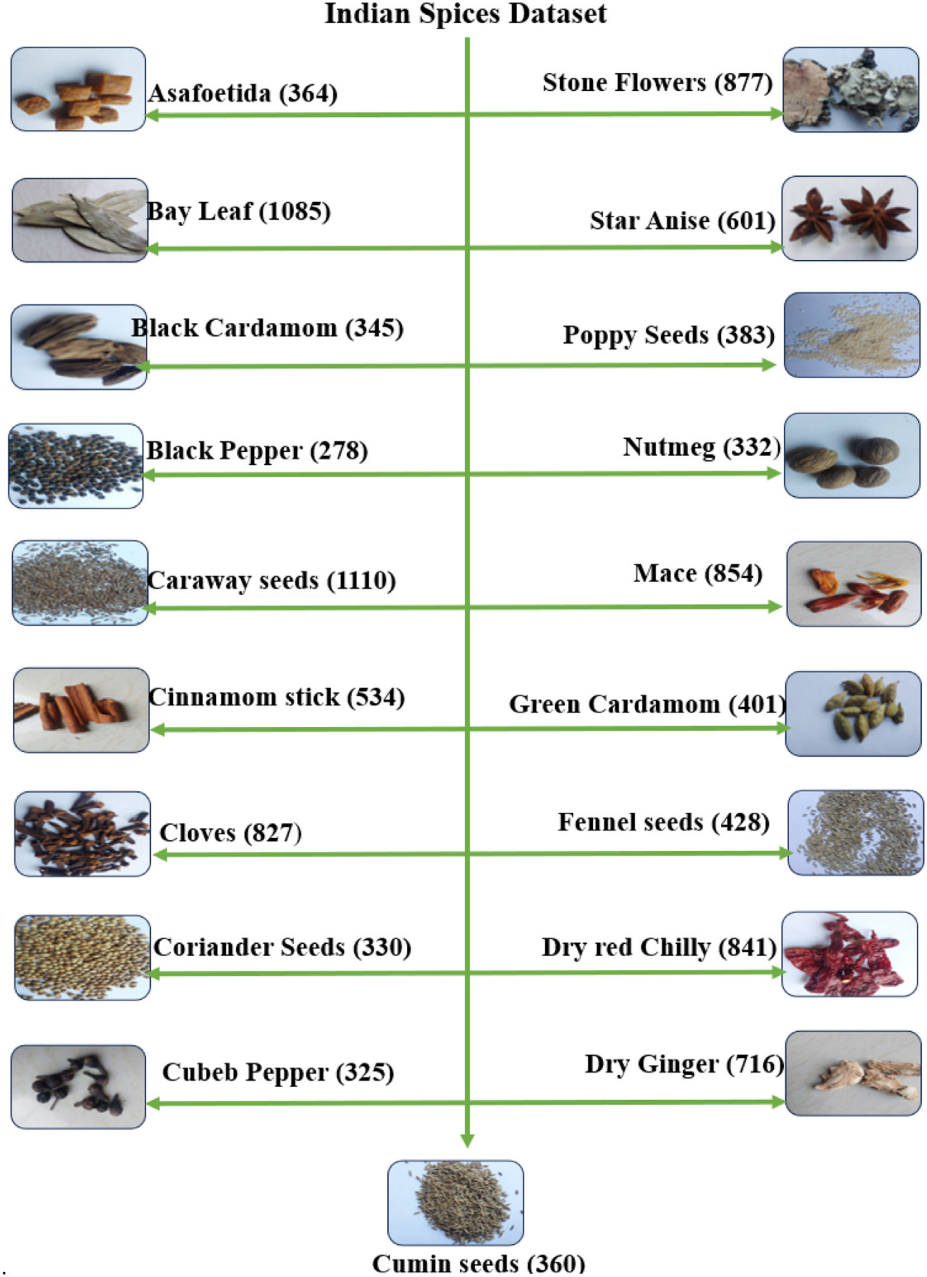
Folder structure of Indian spices dataset.

Table 1 presents a quantitative breakdown of images along with their respective counts. It delineates 19 categories of Indian spices alongside their corresponding image counts. In Fig. 1, the dataset's folder structure is depicted, with spice images displayed within rounded rectangles with their names. The image count for each spice is denoted in brackets beside respective spice image.

**Table 1:** Quantitative breakdown: image count per spice category.

Name of the spices	Count
Asafoetida	364
Bay Leaf	1085
Black Cardamom	345
Black Pepper	278
Caraway seeds	1110
Cinnamom stick	534
Cloves	827
Coriander Seeds	330
Cubeb Pepper	325
Cumin seeds	360
Dry Ginger	716
Dry red Chilly	841
Fennel seeds	428
Green Cardamom	401
Mace	854
Nutmeg	332
Poppy Seeds	383
Star Anise	601
Stone Flowers	877
**Total**	**10,991**

Table 2 provides metadata details for images in the dataset. The metadata fields describe various technical details of an image and its capture settings. The Filename refers to the name of the image file, while ImageWidth and ImageLength indicate the dimensions of the image in pixels. GPSInfo contains geolocation data if available, and ResolutionUnit, XResolution, and YResolution specify the resolution in terms of pixels per unit (inches or centimeters). The ExifOffset points to the location of EXIF metadata within the image file. Camera-specific details include Make and Model, referring to the camera manufacturer and model, while Software refers to any software used to process the image. Orientation specifies the camera's orientation during capture. The DateTime, DateTimeOriginal, and DateTimeDigitized fields provide timestamps for the image's creation and digitization. YCbCrPositioning defines the chrominance positioning, and ExifVersion indicates the version of the EXIF standard used. ComponentsConfiguration provides details about how image components are configured, while ShutterSpeedValue, ApertureValue, and FocalLength describe key camera settings like shutter speed, aperture, and focal length. The BrightnessValue, ExposureBiasValue, MaxApertureValue, and MeteringMode give further insights into the exposure and lighting conditions during capture, and LightSource indicates the type of lighting (e.g., daylight or artificial). he Flash field records whether the flash was used, and ColorSpace defines the color profile in which the image is stored. ExifImageWidth and ExifImageHeight provide the image dimensions as recorded in the EXIF data, while SceneCaptureType specifies the type of scene captured (e.g., landscape, portrait). SubsecTime, SubsecTimeOriginal, and SubsecTimeDigitized capture fractional seconds for more precise timestamps. SensingMethod reveals the type of sensor used in the camera, and ExposureTime and FNumber describe the exposure time and aperture settings. SceneType indicates whether the image was directly captured by a camera or generated, and ImageUniqueID provides a unique identifier for the image. Other fields include ExposureProgram, which defines the exposure mode (manual, automatic, etc.), and ISOSpeedRatings, which records the ISO sensitivity. ExposureMode further specifies if the exposure was manually or automatically set. FlashPixVersion and WhiteBalance describe the FlashPix version and white balance settings used, while FocalLengthIn35mmFilm provides the 35 mm film equivalent for the focal length used during image capture.

**Table 2:** Metadata fields and descriptions for image files.

Filename	ImageWidth	ImageLength	GPSInfo	ResolutionUnit
ExifOffset	Make	Model	Software	Orientation
DateTime	YCbCrPositioning	XResolution	YResolution	ExifVersion
ComponentsConfiguration	ShutterSpeedValue	DateTimeOriginal	DateTimeDigitized	ApertureValue
BrightnessValue	ExposureBiasValue	MaxApertureValue	MeteringMode	LightSource
Flash	FocalLength	ColorSpace	ExifImageWidth	SceneCaptureType
SubsecTime	SubsecTimeOriginal	SubsecTimeDigitized	ExifImageHeight	SensingMethod
ExposureTime	ExifInteroperabilityOffset	FNumber	SceneType	ImageUniqueID
ExposureProgram	ISOSpeedRatings	ExposureMode	FlashPixVersion	WhiteBalance
FocalLengthIn35mmFilm				

Table 3 provides a representative collection of images for the 19 distinct types of Indian spices included in the dataset.

**Table 3:** Sample images of Indian Spices.

**Fig. 2:**
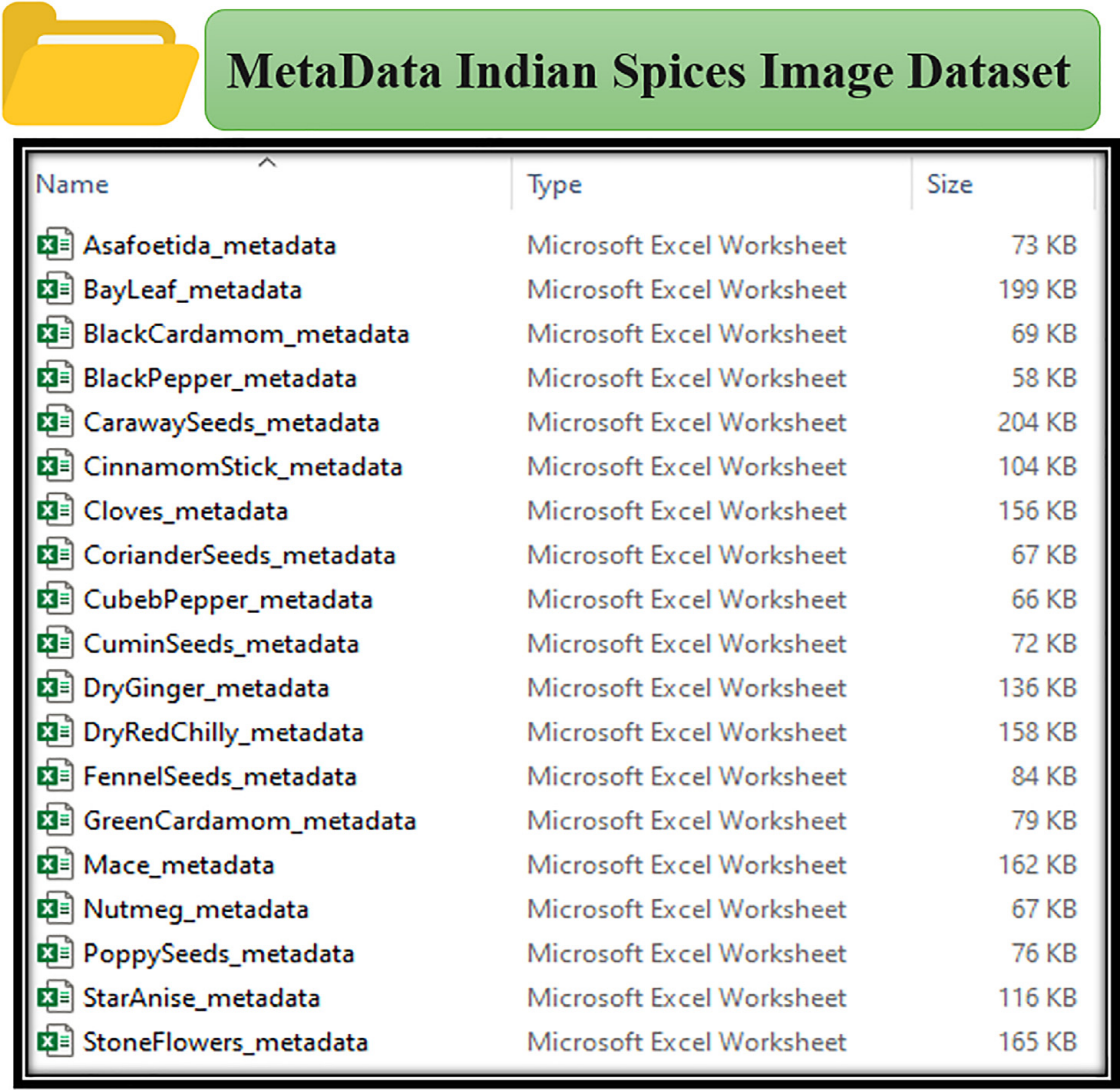
Metadata files for indian spices image dataset.

Fig. 2 shows the collection of metadata files in Excel format for the Indian Spices Image Dataset. Each file corresponds to a specific spice, with metadata extracted for images in individual folders.

## 4 Experimental Design, Materials and Methods

### 4.1 Experimental design

**Fig. 3:**
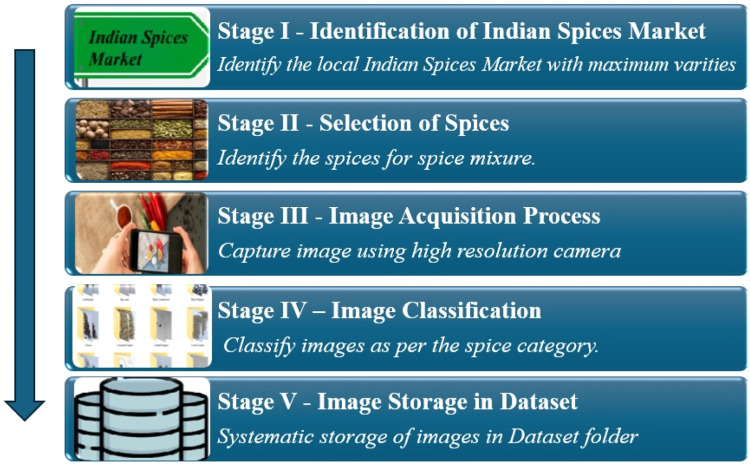
Stage-by-stage progress: Indian spices dataset collection.

This dataset is processed through five stages: Identification of Indian Spices market, Selection of spices, Image acquisition, Image classification and Image storage in dataset. The framework of the proposed pipeline is illustrated in Fig. 3. The processing steps of the Natural Pothole image dataset are described as follows:

• *Identification of Indian Spices Market*: In this stage, research is conducted to identify key marketplaces known for their diversity and authenticity in Indian spices. Market Yard, Gultekadi, Pune, Maharashtra, India, is chosen as a prime location due to its reputation as a major hub for spice trade, ensuring access to a wide range of spices essential to Indian cuisine.
• *Selection of Spices*: Various spices are carefully selected to represent the diverse spectrum of Indian cuisine. A comprehensive list of spices commonly used in Indian cooking is compiled, encompassing both staple and specialty spices. This selection process ensures that the dataset encompasses a broad range of flavors, aromas, and textures characteristic of Indian spices.
• *Image Acquisition*: The image acquisition stage involves capturing high-quality images of the selected spices. Using professional-grade cameras and lighting equipment, the spices are meticulously arranged and photographed from multiple angles with most images taken from the top view to ensure clear visibility of their distinct features. Market Yard serves as the backdrop, providing an authentic setting reflective of the spices' origins. In the quality assurance process for the Indian spices image dataset, images that appeared blurry or had a resolution lower than the specified minimum were rejected to maintain high-quality standards. This ensures that the dataset consists only of clear and detailed images, which are crucial for accurate analysis and machine learning applications. By enforcing these quality criteria, the dataset remains a reliable resource for research and practical use in spice recognition and classification tasks.
• *Image Classification*: Following image acquisition, the dataset undergoes image classification, where each image is labelled with its corresponding spice category. This stage involves manually assigning labels to images based on the spice they represent, ensuring accurate classification for subsequent analysis and model training. IrfanView [16] is a versatile tool used for preprocessing images of Indian spices. It offers batch processing capabilities, allowing users to resize, crop, and convert image formats efficiently, which is essential for managing large images of Indian spices. This ensures uniformity in image size and format across the dataset. Additionally, IrfanView provides basic color correction and enhancement features, improving the visual quality of the images and making them more suitable for analysis and machine learning applications.
• *Image Storage in Dataset*: Once classified, the images are organized and stored in a structured dataset format. Each spice category is allocated a separate folder, and images are named and indexed for easy retrieval and management. The dataset is stored in a standardized format, such as JPG, ensuring compatibility with various image processing and analysis tools. Files are named after the spices, with each image assigned a sequence number. All folders are then compressed using the ZIP format. Every folder is compressed in ZIP format. The ZIP compression format reduces file size using lossless compression and packages multiple files into a single archive. It's widely compatible across platforms, supports encryption, ensures file integrity, and efficiently handles large files, making it ideal for file storage and sharing.

**Fig. 4:**
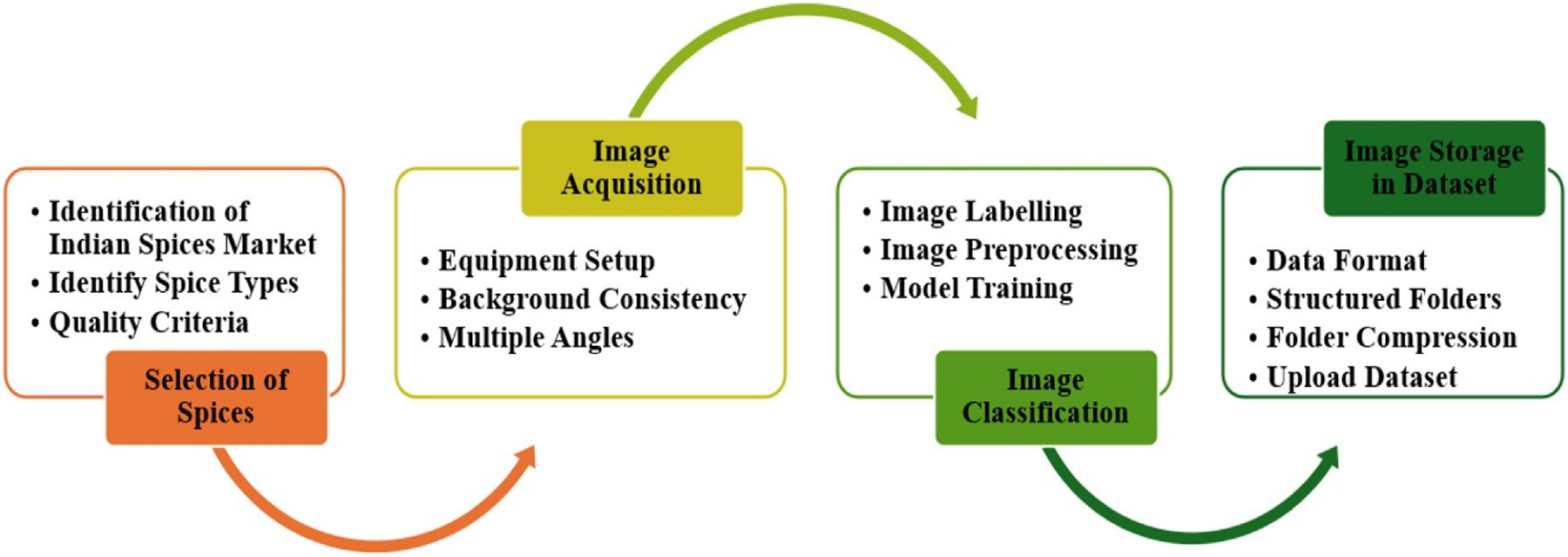
Flowchart of Indian spices dataset generation process.

The generation of an Indian spice's dataset involves several essential steps, as illustrated in Fig. 4, which outlines the flow of execution for creating the dataset. Initially, the selection of spices requires identifying the market and specific types of spices to include, while also establishing criteria to ensure only high-quality samples are chosen for imaging. During the image acquisition phase, high-quality cameras are set up with consistent lighting, and a uniform background, such as a white sheet, is used to maintain consistency across images. Multiple angles are captured, with a focus on top-view images to maximize spice coverage. In the image classification stage, pre-processing steps like cropping, renaming, and resizing images to standard dimensions are performed, followed by the application of machine learning techniques to train classification models. Finally, image storage in the dataset involves selecting an appropriate data format, organizing images into folders, and compressing these folders to facilitate uploading to a dataset repository. This structured approach ensures the creation of a comprehensive and organized dataset of Indian spices.

### 4.2 Materials or specification of image acquisition system

Samsung Galaxy F 23 5G Android Mobile:

• Make and Model: Samsung Galaxy F 23 5G (SM-E236B) Android Mobile.
• Rear Primary Camera: Equipped with a 50-megapixel (f/1.8) lens.
• Camera Sensor: Utilizes the Sony IMX 582 1/2″ sensor.
• Battery: Comes with a 5000 mAh battery.
• Camera Setting: ISO Speed – ISO-40, Maximum aperture:1.69, Exposure bias – 0 Step, Focal Lenth: 5 mm, Light Source: D65, Brightness : 3.75, White Balance: Auto.

During the data collection process, efforts were made to adhere to standardized image acquisition practices, capturing each image using the rear cameras of a Samsung F23 5G Mo- bile known for its high-resolution imaging capabilities. This maintained consistency and quality throughout the dataset. The captured images were saved in JPG format and resized to a resolution of 810 × 1080 pixels.

**Fig. 5:**
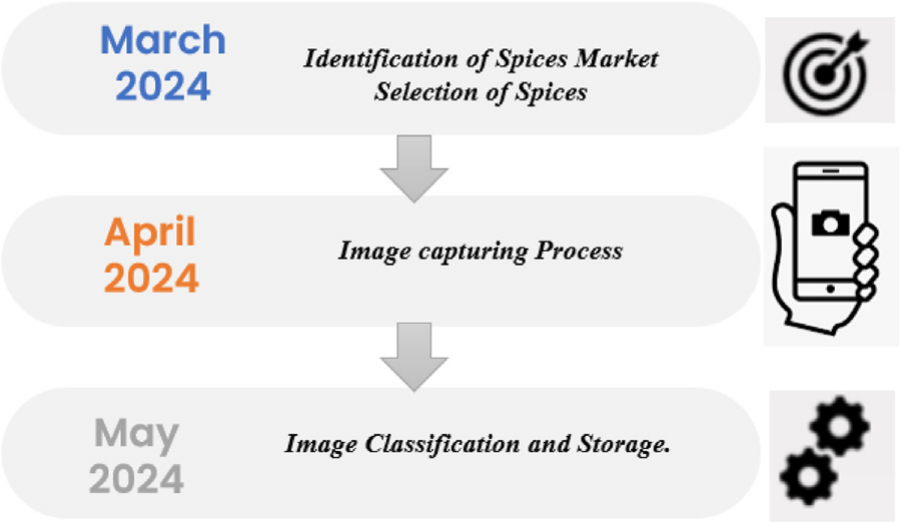
Timeline for the collection of Indian spices dataset.

Fig. 5 shows the timeline for the collection of Indian Spices dataset.

### 4.3 Methods

The creation of the Indian spices dataset follows a structured five-stage process. First, Market Yard in Gultekadi, Pune, Maharashtra, India, was identified for its extensive variety of authentic spices. Then, 19 essential spices, including Asafoetida, Bay Leaf, Black Cardamom, and others, were carefully selected to represent the diversity of Indian cuisine. High-quality images were captured using a high-resolution smartphone camera under controlled lighting conditions, with multiple photographs taken from various angles. These images were manually labelled and classified into categories to ensure accurate and reliable data. Finally, the images were organized into folders based on spice type, saved in JPG format with a resolution of 810×1080 pixels, and systematically stored for easy retrieval and analysis, making the dataset a valuable resource for machine learning, image recognition, and culinary studies.

### 4.4 Evaluation framework

A comprehensive evaluation is essential to determine the dataset's effectiveness in training accurate and reliable models for Indian spices detection. We incorporate key metrics such as accuracy, precision, recall, and the F1-score to provide a holistic understanding of the models' capabilities. Utilizing a dataset of Indian spices images, we employed the VGG16, ResNet50, and InceptionV3 architectures, which are renowned for their proficiency in image recognition tasks. Initially, the model performance was limited, with VGG16 achieving 30 % accuracy, ResNet50 achieving 41 % accuracy, and InceptionV3 achieving 23 % accuracy. However, after training, substantial improvements were observed: VGG16 achieved an accuracy of 90 %, ResNet50 achieved 98 % accuracy, and InceptionV3 achieved 89 % accuracy (Tables 4 and 5).

**Table 4:** Accuracy values for Indian Spices detection model.

Model	Accuracy before Training	Accuracy after Training on our dataset
VGG16	30 %	89.80 %
ResNet 50	41 %	98.23 %
InceptionV3	23 %	88.79 %

**Table 5:** Confusion Matrix Before and after training with different Model.

Confusion matrices on pretrained machine learning models on the dataset before and after training with Indian Spices dataset.

## 5 Dataset Feedback

We welcome user feedback, suggestions, and the sharing of use cases to improve the dataset. Please visit our feedback form at the following URL to contribute your insights and help us enhance this resource.

Dataset Feedback URL: https://forms.gle/KxuN5wKoGDbYqkKK8.

## 6 Github Repo Citation

The Indian Spices Code repository, which provides sample code and resources related to the dataset, is available on Zenodo. It can be cited as follows:

*Kailas, P., Sandip, T., Prawit, C., & Alfa, N. (2024). Indian Spices Code. Zenodo.*https://doi.org/10.5281/zenodo.13640061.

## Limitations

Not Applicable.

## Dataset Availability

The dataset for Indian spices, which includes detailed visual data for research and educational purposes, is available on Mendeley Data [1]. It can be cited as follows:

*Thite, Sandip; Patil, Kailas; Godse, Deepali; chumchu, prawit; Nyandoro, Alfa (2024), “Indian Spices Image Dataset”, Mendeley Data, V2,*10.17632/vg77y9rtjb.3.

## Ethics Statement

Our study does not involve studies with animals or humans. Therefore, we confirm that our research strictly adheres to the guidelines for authors provided by Data in Brief terms of ethical considerations.

## Credit Author Statement

**Sandip Thite:** Conceptualization, Supervision, Writing – review & editing. **Deepali Godse:** Conceptualization, Writing – review & editing. **Kailas Patil:** Methodology, Data curation, Writing – original draft. **Prawit Chumchu:** Writing – review & editing. **Alfa Nyandoro:** Conceptualization, Writing – review & editing.

## Data Availability

Indian Spices Image Dataset (Original data) (Mendeley Data).
